# Three-hour analysis of non-invasive foetal sex determination: application of Plexor chemistry

**DOI:** 10.1186/s40246-016-0066-2

**Published:** 2016-04-04

**Authors:** Adalgisa Pietropolli, Maria Vittoria Capogna, Raffaella Cascella, Chiara Germani, Valentina Bruno, Claudia Strafella, Simona Sarta, Carlo Ticconi, Giusy Marmo, Sara Gallaro, Giuliana Longo, Luigi Tonino Marsella, Antonio Novelli, Giuseppe Novelli, Emilio Piccione, Emiliano Giardina

**Affiliations:** Section of Gynecology and Obstetrics, Academic Department of Biomedicine and Prevention, “Tor Vergata” University Hospital, Oxford Street, 81, 00133 Rome, Italy; Clinical Department of Surgery, “Tor Vergata” University Hospital, Oxford Street, 81, 00133 Rome, Italy; Department of Biomedicine and Prevention, School of Medicine, University of Rome “Tor Vergata”, Montpelier Street, 1, 00133 Rome, Italy; Emotest Laboratory, M. Licola Patria Street, 60, 80078 Pozzuoli, NA Italy; Molecular Genetics Laboratory UILDM, Santa Lucia Foundation, Ardeatina Street, 306, 00142 Rome, Italy; Bambino Gesù Children’s Hospital, IRCCS, Sant’Onofrio Square, 4, Rome, Italy

## Abstract

**Background:**

The knowledge of the individual genetic “status” in the prenatal era is particularly relevant in the case of positive family history for genetic diseases, in advanced maternal age and in the general screening for foetal abnormalities. In this context, here, we report an innovative molecular assay which utilizes the cell-free foetal DNA (cffDNA) as a source for the early and fast detection of the foetal sex. The study involved 132 pregnant women in their first 3 months of pregnancy, who agreed to give a blood sample. All the collected samples were immediately subjected to the separation of the plasma, which was utilized for the extraction of the cffDNA. Successively, the extracted cffDNA was analysed by a quantitative PCR (qPCR) method based on Plexor-HY chemistry, which is able to simultaneously identify, quantify and discriminate the autosomal DNA from the sex-linked DNA.

**Results:**

Overall, the Plexor-HY assay demonstrated to be sensitive and specific for the determination of low-template DNA, such as the cffDNA. In fact, the Plexor-HY assay has been successfully performed in all the samples, identifying 70 males and 62 females. As the foetal sex can be provided in 120 min just by utilizing a maternal blood sample as cffDNA source, the assay represents a very fast, safe and non-invasive prenatal method.

**Conclusions:**

The possibility of determining the foetal sex in the early prenatal life consents the application of our assay as a helpful screening test for subjects and families at risk of sex-linked disorders. Moreover, the early knowledge of the foetal sex may be of great help even for the specialist, who might promptly advise the patients concerning the foetal risk of inheriting sex-linked disorders and the clinical utility of performing an invasive prenatal diagnosis.

**Electronic supplementary material:**

The online version of this article (doi:10.1186/s40246-016-0066-2) contains supplementary material, which is available to authorized users.

## Background

Prenatal diagnosis represents a fundamental step for the identification of genetic pathologies, either caused by numeric and structural chromosomal aberrations, inherited DNA mutations (Mendelian disorders) or malformative parts [1]. In fact, prenatal knowledge of the genetic “status” of an individual is particularly relevant in the case of a positive family history for genetic diseases and advanced maternal age as well as in the general screening of foetal abnormalities [2]. A prenatal diagnosis is essentially composed of two steps: gynaecologic/genetic counselling and gynaecologic/laboratory practice. In particular, gynaecologic/genetic counselling is a service that provides information about the pros and cons of prenatal approaches in terms of the risk, waiting times and reliability of the results [3]. The gynaecologic/laboratory practice includes both prenatal techniques (invasive and non-invasive) and genetic approaches (karyotyping and molecular assays) [4]. To date, the invasive approaches (amniocentesis and chorionic villous sampling, CVS) are considered to be the gold standard techniques for prenatal testing, although these procedures can sometimes be fatal for the foetus (~0.2–0.5 %) [5, 6].

The non-invasive prenatal tests (NIPTs) allow for the gathering of information about the “status” of the foetus without affecting the structures that are primarily involved in foetal development/life [7]. The NIPT methods are therefore safer in terms of abortion risk relative to the invasive methods. The NIPTs can be distinguished in diagnostic (ultrasonography) and non-diagnostic (biochemical screening and the analysis of the cell-free foetal DNA (cffDNA)) approaches [8, 9].

Analysis of cffDNA is the most recent non-diagnostic NIPT method. Although the source of cffDNA is not completely understood, it may originate from fragments of apoptotic placental cells (trophoblast cells) [10]. The cffDNA is highly detectable in the maternal blood, either in the plasma or in serum fractions at the fifth week of pregnancy [11, 12]. The concentration of cffDNA increases during the weeks of gestation and ranges from 3.3 to 69.4 to 76.9 to 769 copies/ml (accounting for 3 to 6 % of the free DNA present in the maternal circulation [13]. Given its characteristics, cffDNA represents as an excellent source of DNA that is applicable to the early prenatal tests [12]. On this subject, many innovative next-generation sequencing (NGS) platforms have been recently designed to evaluate chromosomal or DNA alterations with massive and high-throughput approaches. In addition, the NGS techniques are also able to discriminate foetal sex with high-accuracy and sensitive results. However, the effective application of NGS in clinical practice is limited by elevated costs in terms of the time and equipment required for the whole analysis [14]. Sex determination is, in fact, usually performed through the application of PCR-based techniques, either standard PCR or real-time PCR [15].

Knowledge of foetal sex can provide helpful information on the risk of X-linked diseases (Duchenne’s dystrophy, fragile X disorder and haemophilia), metabolic conditions associated with development of ambiguous external genitalia and risk of congenital adrenal hyperplasia [15–17].

In this context, we describe a 3-h molecular assay that utilizes cffDNA as a source for the early detection of foetal sex.

## Methods

This study was previously approved by the ethics committee of the University of Rome “Tor Vergata”. The study was performed according to the tenets of the Declaration of Helsinki. The samples were recruited from the Department of Biomedicine and Prevention, Section of Obstetrics and Gynaecology at “Tor Vergata” University Hospital Rome, Italy. All of the participants were also subjected to invasive prenatal diagnosis (IPD) through traditional methods, in particular, amniocentesis or CVS (indications for IPD were advanced maternal age or abnormal results of NIPTs). The clinical data of the recruited samples are reported in Additional file 1. Signed informed consent was obtained from all of the participants before the blood sample collection. Peripheral blood samples (10 ml) were collected from 132 pregnant women during their first 3 months of pregnancy (from the 12th to 14th gestational weeks, in particular 44 women per class). The collected samples were utilized for the extraction of the cffDNA.

The cffDNA was analysed with a fluorescence-based quantitative PCR (qPCR) method to simultaneously identify, quantify and discriminate the autosomal DNA from the sex-linked DNA. The qPCR procedure was performed utilizing Plexor-HY chemistry (Promega), which exploits specific primers for the amplification of Y-chromosomal DNA, human autosomal DNA and an internal positive control (IPC). Additionally, the Plexor-HY assay provided a “standard DNA”, which is a synthetic DNA (concentrated to 50 ng/μl) that is necessary to quantify the unknown samples (through the realization of a “standard curve” obtained by the serial dilution of the standard DNA from 10 to 0.032 ng/μl).

The specificity and reliability of the qPCR reaction is assessed by the generation of a dissociation curve and the calculation of the melting temperature (*T*_m_) of the products after the amplification [18]. In our study, the dissociation curve and the *T*_m_ were crucial for the interpretation of the results and the correct determination of foetal sex.

The foetal sex of the samples was established according to the presence/absence of a fluorescence signal, which was emitted only in the presence of a Y-chromosomal DNA substrate. Therefore, the only sex-linked DNA that can be quantified is from the Y-chromosomal DNA and it is correlated with the amount of emitted-fluorescence. The quantity of X-chromosomal DNA cannot be specifically detected because the Plexor-HY system does not contain the primers for its amplification. The female sex is thereby deducible on the basis of the missing fluorescence (instead of the detection of a fluorescent signal) as well as of a “flat” dissociation curve (due to the absence of amplification). This aspect actually represents a limit of the method, which can be partially overcome by comparing the unknown samples with the control samples (with known sex).

Concerning the amplification/quantification of the autosomic DNA, it can be performed with all of the samples independently from gender.

All the technical details of the cffDNA extraction and of the Plexor-HY assay for the foetal sex determination have been reported in the Additional file 2.

## Results

**Fig. 1:**
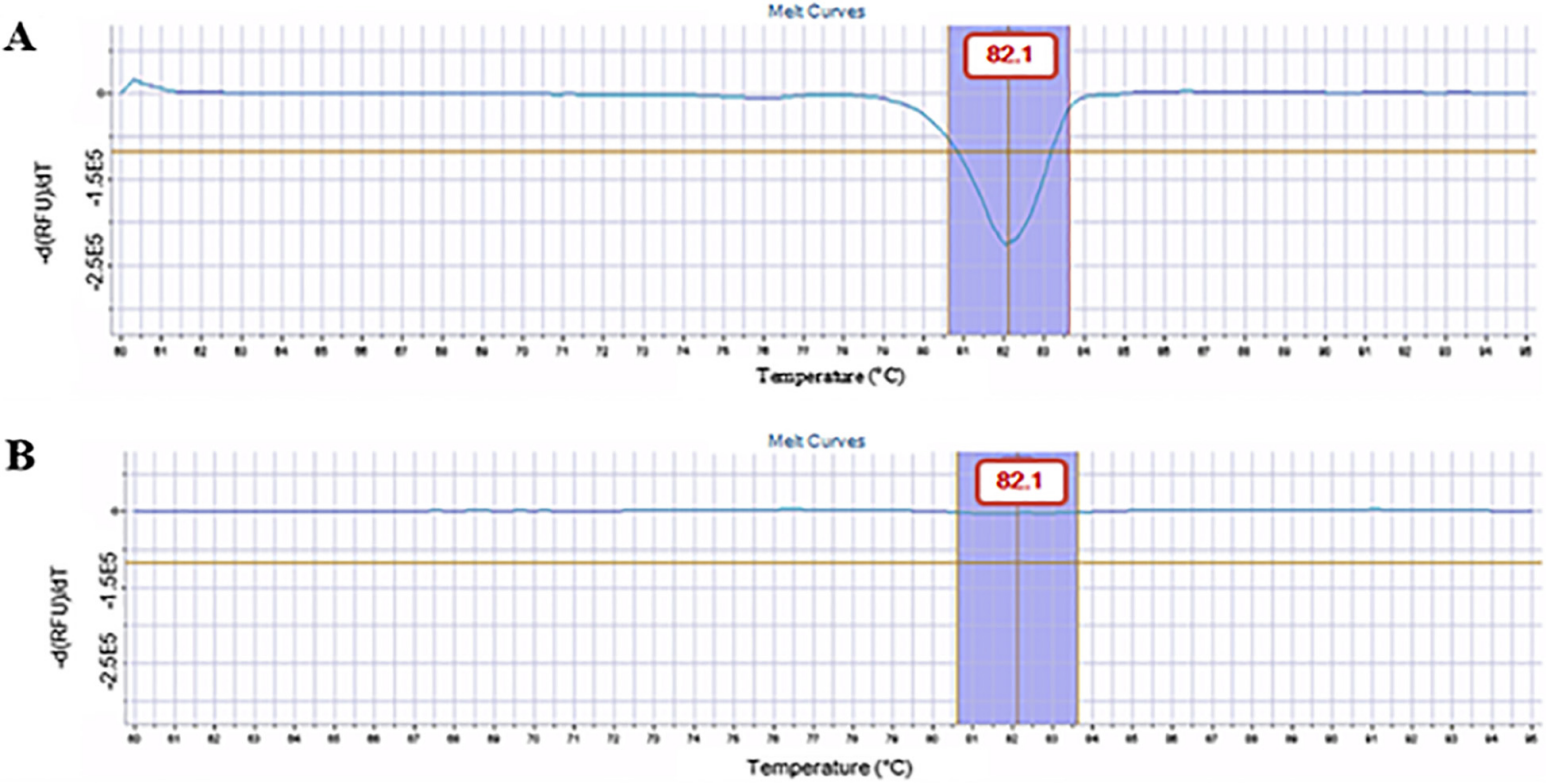
**a** Result interpretation of the Plexor-HY assay on cffDNA. The detection of the fluorescence signal is indicative of the presence of Y-chromosomal DNA substrate (male foetal sex). **b** Result interpretation of the Plexor-HY assay on cffDNA. In this case, the absence of the fluorescence signal is interpreted as the presence of X-chromosomal DNA substrate (female foetal sex)

The molecular assay for the simultaneous determination of foetal sex and quantification of the autosomic/sex-linked DNA has been successfully performed in all 132 samples, identifying 70 males and 62 females. Figure 1a represents the melting curve from the amplification of the Y-chromosomal DNA (male foetal sex) sample with its specific *T*_m_ (82.1 °C) and fluorescence intensity. Figure 1b depicts the “flat” dissociation curve resulting from the absence of a fluorescent signal (no amplification) and indicates the presence of X-chromosomal DNA (female foetal sex).

Our method has been validated in terms of specificity and sensitivity for the determination of foetal sex by comparing our results with those obtained from traditional prenatal tests (i.e., amniocentesis, CVS and ultrasonography) on the same samples. The comparison between Plexor-HY technology and the common procedures showed a 100 % concordance, with no false positive/negative results (Table 1). In addition, the sensitivity of the Plexor-HY assay has been evaluated by the calculation of the minimum detectable concentration (limit of detection (LOD)) of cffDNA. The LOD has been measured testing cffDNA concentrations included in a range from 10 to 0.0001 ng/μl. These experiments showed that the LOD for Plexor-HY assay is 0.0001 ng/μl ± 0.5.

**Table 1:** Concordance analysis of the foetal sex detected by cffDNA and the traditional prenatal approaches

Gestational week (w^a^) of blood sampling	cffDNA^b^	CVS^c^	Amniocentesis	Concordance (%)
	(Foetal sex)	(Foetal sex)	(Foetal sex)	
12	44	32	12	100
	(M^d^ = 24; F^e^ = 20)	(M = 17; F = 15)	(M = 7; F = 5)	
13	44	20	24	100
	(M = 22; F = 22)	(M = 8; F = 12)	(M = 14; F = 10)	
14	44	9	35	100
	(M = 24; F = 20)	(M = 5; F = 4)	(M = 19; F = 16)	

^a^Gestational week

^b^Cell-free foetal DNA

^c^Chorionic villous sampling

^d^Males

^e^Females

As expected, the amount of autosomic/sex-linked cffDNA was found to be increased from the 12th to the 14th gestational week. In fact, the autosomic DNA amount ranged from 1.42 × 10^−2^ to 1.86 × 10^−1^ pg/μl, while the sex-linked DNA (Y-chromosomal DNA) concentration varied from 0.009 to 0.09 pg/μl throughout the gestational weeks taken into consideration.

## Discussion

Overall, the Plexor-HY assay was shown to be sensitive and specific for the determination of low-template DNA, such as cffDNA. Despite other qPCR methods, Plexor chemistry based on the incorporation of modified nucleotides, multicopy gene targets and generation of a dissociation curve made the assay highly performant and reliable for the analysis of cffDNA.

**Fig. 2:**
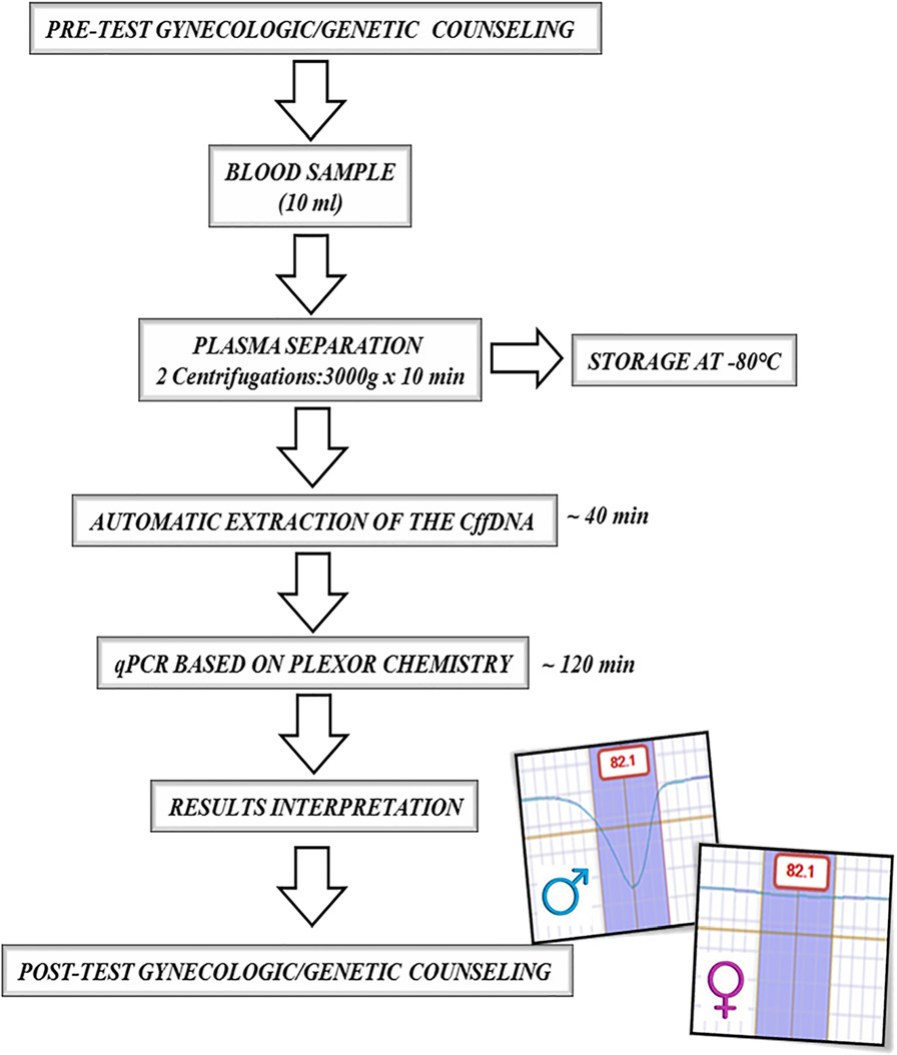
Workflow for the foetal sex determination by the Plexor-HY assay on cffDNA

As the foetal sex can be determined within 120 min by utilizing a maternal blood sample as a DNA source, the assay is a very fast, safe and non-invasive prenatal method. In Fig. 2, the workflow depicts the different operational phases (counselling and laboratory practice) that characterize the test for early foetal sex determination by our method.

Given the possibility of applying the Plexor-HY assay for the identification of foetal sex in the first trimester (12th gestational week), it may represent a helpful screening test for inherited X-linked disorders, such as Duchenne’s muscular dystrophy (DMD). The DMD is an X-linked recessive disorder with an incidence of 1/3300 male newborns. The clinical picture of the disease is characterized by the atrophy and progressive degeneration of the muscular fibres that lead to a deficit of muscular strength in the first 3–5 years of life. Concerning the genetic picture of the disease, several mutations in the DMD gene (Xp21.2, codifying for the dystrophin protein) have been identified as major causes of the pathologic phenotype.

On this subject, we evaluated the clinical utility of the Plexor-HY System in two families with a positive history for DMD. The blood samples of the two pregnant women were taken at their 12th gestational week to extract and analyse the cffDNA. The application of the Plexor-HY technology led to the identification of one female foetus and one male foetus, respectively. Given these results, the early prenatal identification of the foetal sex was useful to provide an initial indication about the risk of inheriting DMD. The results obtained by the Plexor-HY assay have been successively confirmed with the DNA analysis performed by an invasive procedure (CVS). These results suggested that our method may be employed to reassure patients about the risk of inheriting DMD in relation to foetal sex and eventually avoid further invasive procedures. However, it is important to remind that the test is able to determine the foetal sex, while the effective diagnosis of DMD can only be done by the traditional molecular diagnostic approaches.

The determination of foetal sex by non-invasive cffDNA analysis may be thus validated as a rapid, safe and early prenatal screening test for subjects and families at risk of X-linked disorders, metabolic condition associated with ambiguous development of external genitalia and risk of congenital adrenal hyperplasia. This approach may be really advantageous for both the woman and the foetus, since the risk of abortion caused by the traditional invasive techniques is effectively abolished.

In this context, genetic counselling plays a fundamental role in providing the right support to the patient, both in the pre- and post-test phases. In fact, counselling before the test is necessary to elucidate the utility of the early prenatal screening of foetal sex, while post-test counselling has to explain the result of the test to enable the patient/couple to consciously take her/their own decision.

In conclusion, here, we report a 3-h molecular assay that utilizes cffDNA as the source for the early detection of the foetal sex. Our results showed that Plexor chemistry with cffDNA is a reproducible, sensitive, robust and non-invasive method for prenatal screening of the gender. Moreover, the early knowledge of foetal sex may be of great help even for the specialist, who might promptly advise the patients about the risks (sex-linked disorders) for the foetus and the clinical utility of performing an invasive prenatal diagnosis.

### Ethics approval and consent to participate

The study has been approved by the Ethics Committee of the University of Rome “Tor Vergata”. The study was performed according to the tenets of the Declaration of Helsinki.

### Availability of data and materials

The datasets supporting the conclusions of this article are available in the main paper.

### Consent

The participants have signed the informed consent for publication of personal data.

## Additional files

Additional file 1: Table S1. Major clinical characteristics of study women (*n* = 132). (DOCX 12.5 kb) Additional file 2: Technical details of the Plexor-HY method for the foetal sex determination. (DOCX 14.7 kb)

## Abbreviations

cffDNA: cell-free foetal DNA
CVS: chorionic villous sampling
DMD: Duchenne’s muscular dystrophy
IPD: invasive prenatal diagnosis
NGS: next-generation sequencing
NIPTs: non-invasive prenatal tests
qPCR: quantitative PCR
*T*_m_: melting temperature
