# Supplementary material for: Three-hour analysis of non-invasive foetal sex determination: application of Plexor chemistry
Source: Hum Genomics. 2016 Apr 4;10:9. doi: 10.1186/s40246-016-0066-2 (PMC4820952; doi:10.1186/s40246-016-0066-2)
Supplement: Additional file 1: Table S1. — Major clinical characteristics of study women (n = 132). (DOCX 12.5 kb) [file 40246_2016_66_MOESM1_ESM.docx]

**S 1. Major clinical characteristics of study women (n = 132).**

Data are expressed as mean + SD or percentage.

| Clinical Characteristics | + SD^a^ or percentage |
| --- | --- |
| Age (years) | 36.2 + 4.3 |
| Caucasian race (%) | 132 (100 %) |
| BMI^b^ (Kg/m^2^) | 23.9 + 12.2 |
| Present smokers (%) | 5 (6.6%) |
| Spontaneous pregnancy (%) | 132 (100 %) |
| Singleton pregnancy (%) | 132 (100 %) |
| Nulliparous women (%) | 132 (100 %) |
| ^a^ Standard Deviation  ^b^ Body Mass Index |  |
