# Supplementary material for: Three-hour analysis of non-invasive foetal sex determination: application of Plexor chemistry
Source: Hum Genomics. 2016 Apr 4;10:9. doi: 10.1186/s40246-016-0066-2 (PMC4820952; doi:10.1186/s40246-016-0066-2)
Supplement: Additional file 2: — Technical details of the Plexor-HY method for the foetal sex determination. (DOCX 14.7 kb) [file 40246_2016_66_MOESM2_ESM.docx]

**Technical details of the Plexor-HY method for the foetal sex-determination.**

The collected blood samples were immediately processed for the isolation of the plasma and extraction of the cffDNA. The plasma was separated by performing an initial centrifugation of the blood samples at 3000 g for 10 minutes. Successively, an additional centrifugation (at 3000 g for 10 minutes) of the plasma was performed to obtain a clean and high-quality sample. The separated plasma can be stored at -80°C for long periods of time or can be directly utilized for cffDNA extraction. In particular, the cffDNA was extracted from 1ml of each plasma sample with the extraction kit “Maxwell® 16 Circulating DNA Purification” (according to the manufacturer’s instructions) and the AS2000 Maxwell® 16 automatic extractor (Promega). This automated approach took only 40 minutes to extract the cffDNA through its magnetic bead technology.

The cffDNA was analysed with the qPCR-based Plexor-HY assay (Promega). One of the key points of the method lies in the careful design of the primer sequences that recognize a multicopy gene target. In particular, one of the primers for autosomic DNA and one for the sex-linked DNA contain a modified nucleotide at its 5’ end (5′-methylisocytosine, iso-dC), and both were labelled with a fluorescent molecule (reporter). The other two primers for autosomic and sex-linked DNA amplification instead are unlabelled. The reaction mix also includes some modified nucleotides (e.g., isoguanine and iso-dG), which have a quenching molecule (dabcyl, quencher) enclosed. Before the extension phase of the qPCR procedure, the reporter is far from the quencher, so that it is able to emit a fluorescent signal. During the extension step of qPCR, the modified nucleotides are incorporated together with the quencher, which comes in close proximity to the reporter and causes shielding of the fluorescent signal (emitted by the reporter). The interaction between the reporter and quencher during the amplification thereby results in a decrease of the fluorescence in proportion to the accumulation of the PCR product.

All of the samples were analysed in triplicate to verify the reproducibility of the results. The final PCR volume was 20 μl and included: 1.0 μl of Plexor® HY 20X Primer, 10 μl of Plexor® HY Master Mix, 7.0 μl of amplification grade water, and 2.0 μl of cffDNA and DNA Standard. The PCR cycle was composed of two steps at 95°C for 2 min followed by 95°C for 5 sec and 60°C for 35 sec (the last two steps were repeated for 38 cycles). The specificity of the reaction was assessed by generating a melting curve through the incubation of the PCR products at 95°C for 15 sec, 60°C for 1 min and 95°C for 15 sec. The qPCR reactions procedures were performed on a 7500 Fast Real-Time PCR System (Applied Biosystems), while the output data were analysed with Plexor™ Analysis Software (Promega).

The efficiency of the qPCR reaction have been further confirmed and monitored because of the presence of positive (samples with known sex) and negative (samples with no template DNA) control samples.
